# JAK2 activation promotes tumorigenesis in ALK-negative anaplastic large cell lymphoma via regulating oncogenic STAT1-PVT1 lncRNA axis

**DOI:** 10.1038/s41408-021-00447-x

**Published:** 2021-03-12

**Authors:** Kang Le, Linda E. Wellik, Matthew J. Maurer, Ellen D. McPhail, Thomas E. Witzig, Mamta Gupta

**Affiliations:** 1 Department of Biochemistry and Molecular Medicine, School of Medicine and Health Sciences, GW Cancer Center, Washington, DC USA; 2 Division of Hematology, Mayo Clinic Rochester, Rochester, MN USA; 3 Department of Health Sciences, Mayo Clinic Rochester, Rochester, MN USA; 4 Department of Laboratory Medicine, Mayo Clinic Rochester, Rochester, MN USA

**Keywords:** Haematological diseases, Haematological cancer

Dear Editor,

Anaplastic large cell lymphoma (ALCL), a subgroup of peripheral T-cell lymphoma (PTCL), is characterized by high expression of CD30 with varying genetics and clinical outcome^1^. Studies confirm that the 5 years overall survival rates for ALK-positive and ALK-negative ALCL at 80% and 49%, respectively^2,3^. Inferior clinical outcome in patients with ALK-negative ALCL as compared to ALK-positive ALCL highlights the need for new druggable targets for this unique subgroup of ALCL. Prior studies have demonstrated upregulated JAK kinases and their downstream STAT-signaling proteins in various subtypes of PTCLs^4–8^; however, studies delineating the role of JAK isoforms in ALK-negative ALCL are lacking. The overall goal of this study was to assess role of JAK kinases in the ALK-negative ALCL subset and to evaluate the potential impact on disease prognosis and therapeutic advances.

We performed immunohistochemistry on ALK-negative tumors (*n* = 12) using phospho-specific antibodies against JAK1, JAK2, and JAK3. Using 30% cut-off, ALK-negative ALCL tumors were negative for p-JAK1;10% positive for p-JAK3, while 33.3% showed elevated p-JAK2. Consistent with the p-JAK2 immunostaining seen in ALK-negative ALCL, robust tyrosine phosphorylation of JAK2 was detected in Mac-2A and FE-PD cells (Fig. 1A; S1A, B). These results suggest that JAK2 activation is exclusively associated with the ALK-negative ALCL subtype, and may have an important role in the disease pathogenesis. Pharmacologic inhibition of JAK2 specific inhibitor fedratinib, resulted in a dose-dependent decrease in cell proliferation and survival in Mac-2A and FE-PD cells in vitro. Similarly, shRNA-mediated knockdown of JAK2 significantly (*p* < 0.01) decreased the growth of Mac-2A cells. Furthermore, inhibition of JAK2 abated colony formation capabilities of the FE-PD and Mac-2A cells (Fig. 1B–D; S1C). Taken together, these results suggest that ALK-negative ALCL cells have an exquisite sensitivity to JAK2 inhibition. In order to find out the downstream mediators of JAK2 kinase in the ALK-negative ALCL, effects of fedratinib or JAK2 siRNA were assessed on the STATs signaling. Treatment with fedratinib decreased the phosphorylation of STAT1 and STAT3 in Mac-2A and FE-PD cells. Similarly, JAK2 knockdown by siRNA also decreased phosphorylation of STAT1 and STAT3 in Mac-2A cells (Fig. 1E; S2A). Interestingly, fludarabine (a STAT1-inhibitor) but not stattic (a-STAT3 inhibitor) had a significant (*p* < 0.01) inhibitory effect on cell proliferation and survival of Mac2A and FE-PD cells (Fig. 1F; S2B). STAT1 depletion by STAT1-shRNA significantly (*p* < 0.01) inhibited the proliferation of FE-PD cells (Fig. S2C, D). The CFU assay also demonstrated that the STAT1 inhibition with fludarabine or STAT1 shRNA significantly inhibited the colony-forming ability of ALK-negative ALCL cells (Fig. S2E). These data suggest that STAT1 is an important downstream mediator of oncogenic effects of JAK2 in the ALK-negative ALCL cells.

**Fig. 1:**
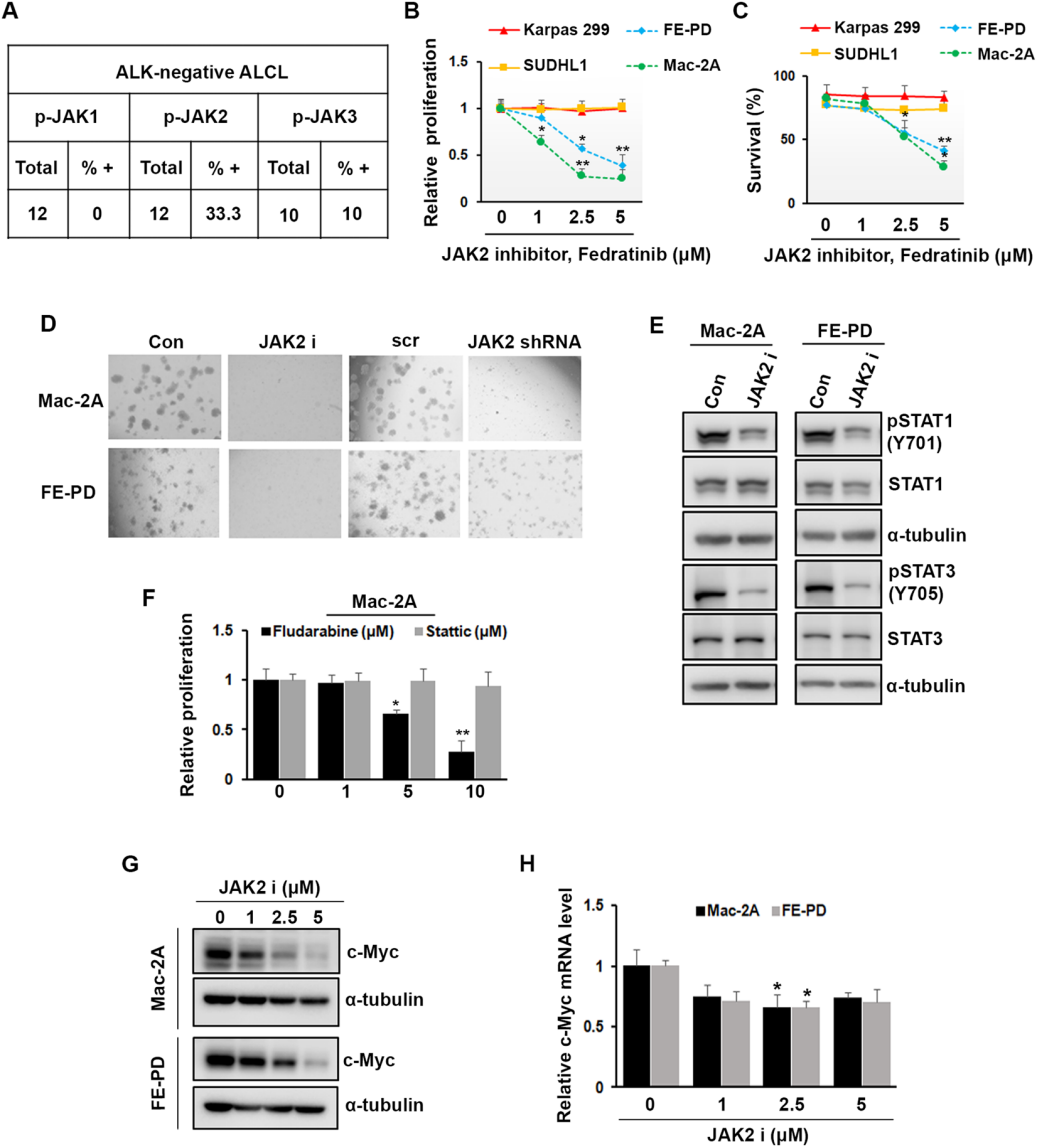
JAK2 regulates ALK-negative ALCL cell growth via STAT1/c-Myc signaling. **A** Summary of pJAK1, pJAK2, and pJAK3 staining by IHC in ALK-negative ALCL tissues by IHC. **B**–**C** ALK-negative (Mac-2A and FE-PD) and ALK + ALCL cell lines (Karpas 299 and SUDHL1) were treated with the indicated fedratinib, and proliferation/survival was assessed (*P < 0.05, **P < 0.01). **D** Mac-2A and FE-PD cells were plated in semisolid Metho-Cult medium left untreated and treated with fedratinib or JAK2 shRNA, and the colony numbers were scored after 10 days. **E** Mac-2A and FE-PD cells were treated with fedratinib (2.5 μM) for 24 h and then blotted with pSTAT1 (Tyr701) and pSTAT3 (Tyr705) antibodies. **F** Mac-2A cells were treated with the indicated concentration of fludarabine (STAT1 inhibitor) or Stattic (STAT3 inhibitor) for 72 h and proliferation was assessed by MTT assay (*P < 0.05, **P < 0.01). **G**–**H** Mac-2A and FE-PD cells were treated with indicated concentration of fedratinib for 48 h and protein level of c-Myc (**G**) and mRNA level of c-Myc (**H**) was assessed by western blotting and QRT-PCR respectively (*P < 0.05).

We and others have previously shown that c-Myc (referred as Myc hereafter) plays an important role in the lymphoma subtypes and acts as an important downstream effector to JAK/STAT pathway^9–11^. In line with this notion, inhibition of JAK2 with fedratinib decreased Myc at protein level whereas mRNA level or distribution pattern between cytoplasm and nucleus remained unaltered in both Mac-2A and FE-PD cell lines. A rapid decline in Myc protein levels after JAK2 inhibition suggests that JAK2 might regulate the stability of Myc protein by post-translational modifications. In a follow-up experiment, proteasome inhibitor-MG132 prevented Myc degradation following JAK2 inhibition in ALK-negative cell line (Fig. 1G, H; S2F, G). In addition, the ubiquitination assay demonstrated that fedratinib effectively facilitates the poly-ubiquitination modification of Myc protein in Mac-2A cells (Fig. S2H). These results indicate that JAK2 regulates Myc protein stability in ALK-negative ALCL cells.

Since Myc translocations are not reported in the PTCL, we speculate that ALK-negative ALCL may have distinct molecular mechanisms that drive Myc expression. It has been recently shown that Myc partners with the adjacent long non-coding RNA (lncRNA) PVT1, which stabilizes Myc protein in some cancers^12^. We have seen that lncRNA-PVT1 was highly expressed in FE-PD and Mac-2A compared with normal CD3+ T cells and located in both the cytoplasmic and nuclear fractions. Furthermore, silencing PVT1 expression by siRNA facilitated Myc protein degradation that was rescued by proteasome inhibitor MG132 suggesting an important role of PVT1 in the stabilization of Myc protein in ALK-negative ALCL (Fig. S3A–C). RNA-immunoprecipitation (RNA-IP) using an anti-Myc antibody showed that PVT1 lncRNA (but not MALAT1 or NEAT1 lncRNAs) can directly bind with Myc protein in FE-PD cells, and this interaction was abolished when JAK2 is inhibited (Fig. 2A, B). To further investigate the regulatory mechanism underlying JAK2-STAT1, we analyzed PVT1 promoter sequence and discovered three putative STAT1-binding fragments or elements (F1, F2, and F3) based on JASPAR database. Chromatin immunoprecipitation assays confirmed STAT1 binding to the F3 site of the PVT1 promoter (Fig. 2C, D). Furthermore, silencing STAT1 expression using two independent shRNA constructs significantly (*p* < 0.05) inhibited the PVT1 transcript level, although the effect of #2 shRNA was relatively more profound than #1 shRNA. A similar inhibitory effect was observed with STAT1 specific inhibitor on PVT1 expression in the Mac-2A and FE-PD cells (Fig. S3D, E). Taken together, these data suggest that PVT1 may be a transcriptional target of JAK2/STAT1 signaling in ALK-negative ALCLs. Next, we sought to determine whether PVT1-lncRNA is interacting with STAT1 and providing a feedback loop for continuous oncogenic effect. RNA-IP showed that PVT1 (but not MALAT1-lncRNA) was detected in the pSTAT1 and total STAT1 immunoprecipitated RNAs in the FE-PD cells. Phosphorylation of STAT1 significantly decreased after PVT1 depletion with siRNA #2, however total STAT1 protein level did not change (Fig. 2E; S3F, G), suggesting that PVT1 might regulate STAT1 activity without regulating STAT1 protein stability in the ALK-negative cells. As expected, JAK2 inhibition inhibited STAT1 and PVT1 interaction in the FE-PD and Mac-2A cells assessed by RNA-IP. Similarly, combined effect of JAK2 inhibitor in the STAT1 inhibited FE-PD cells was more profound on the expression of Myc protein than either agent alone (Fig. 2F; S3H).

**Fig. 2:**
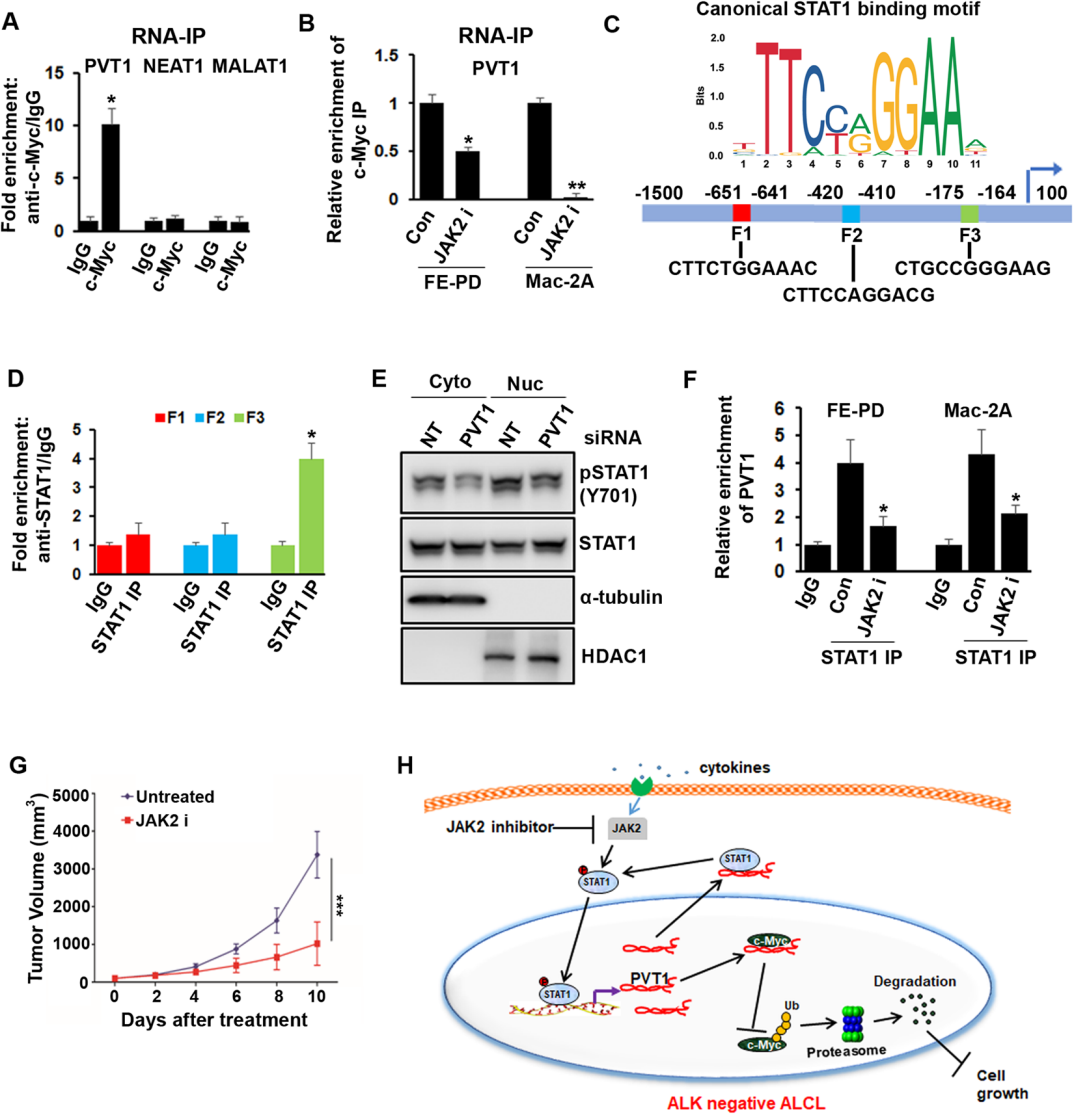
Involvement of STAT1 and PVT1 lncRNA feedback loop in ALK-negative ALCL. **A** RNA-IP was performed in the lysate of FE-PD cells using c-Myc antibody and QRT-PCR was performed using specific primers for PVT1, NEAT1, and MALAT1 lncRNAs (*P < 0.05). **B** RNA-IP was performed in the Mac-2A and FE-PD lysate treated with fedratinib and QRT-PCR was performed using PVT1 primers (*P < 0.05, **P < 0.01). **C** Schematic diagram of STAT1-binding motif and three potential STAT1 responsive elements (F1, F2, and F3) in the PVT1 promoter region. **D** The enrichment of STAT1 on PVT1 promoter (F1, F2, and F3) was detected by chromatin immunoprecipitation assay (*P < 0.05). **E** Mac-2A cells were incubated with non-target (NT), or specific PVT1 siRNA for 72 hours before analysis of indicated proteins in the cytoplasmic and nuclear fractions assessed by western blotting. **F** QRT-PCR of STAT1 enriched PVT1 lncRNA following immunoprecipitation with anti–STAT1 antibody in the presence and absence of JAK2 inhibitor fedratinib (*P < 0.05). **G** In vivo fedratinib treatment decreased tumor volume as compared to the untreated group (***P < 0.001). **H** Schematic diagram of JAK2 regulating ALK-negative ALCL cell growth via STAT1-PVT1-Myc signaling cascade.

We next investigated the efficacy of JAK2 inhibitor fedratinib, using a xenograft model established from subcutaneously injected FE-PD cell line in 6-8 weeks old NOD/SCID (The Jackson Laboratory, ME, USA) mice (*n* = 10). Daily dosing with 100 mg/kg fedratinib for 10 days led to significant suppression of tumor growth as compared to that in vehicle-treated animals. Fedratinib in vivo treatment was well tolerated with no significant weight loss experienced over the course of treatment (Fig. S4A–C). Fedratinib treatment significantly decreased tumor volume and tumor weight (*p* *<* 0.001) as compared with the untreated group. (Fig. 2G; S4D). Interestingly, Myc mRNA and PVT1 lncRNA levels were significantly low in tumors harvested from animals treated with fedratinib as compared to those from vehicle-treated animals. Similarly, decreased expression of pSTAT1 and Myc protein was detected in tumors harvested from fedratinib treated group as compared to the control group (Fig. S4E–G). A schematic diagram showing JAK2 mediated function in pJAK2 over-expressing ALK-negative ALCL is shown (Fig. 2H).

Taken all together, here we demonstrate that in ALK-negative ALCL cells, JAK2 is activated and its inhibition inhibited tumorigenesis via STAT1-PVT1- Myc axis. Beyond the potential therapeutic significance of constitutively activated JAK2, our study provides mechanistic insights that JAK2 potentially contributes to the pathogenesis of a subsets of ALK-negative ALCL. Based upon these finding our results suggest that the PVT1-lncRNA expression may be a useful biomarker for the prognosis, and targeting JAK2 would be a reasonable treatment strategy to patients with ALK-negative ALCL.

## Supplementary information

Supplementary Figure 1

Supplementary Figure 2

Supplementary Figure 3

Supplementary Figure 4
