# Supplementary figures and images for: JAK2 activation promotes tumorigenesis in ALK-negative anaplastic large cell lymphoma via regulating oncogenic STAT1-PVT1 lncRNA axis
Source: Blood Cancer J. 2021 Mar 12;11(3):56. doi: 10.1038/s41408-021-00447-x (PMC7955124; doi:10.1038/s41408-021-00447-x)

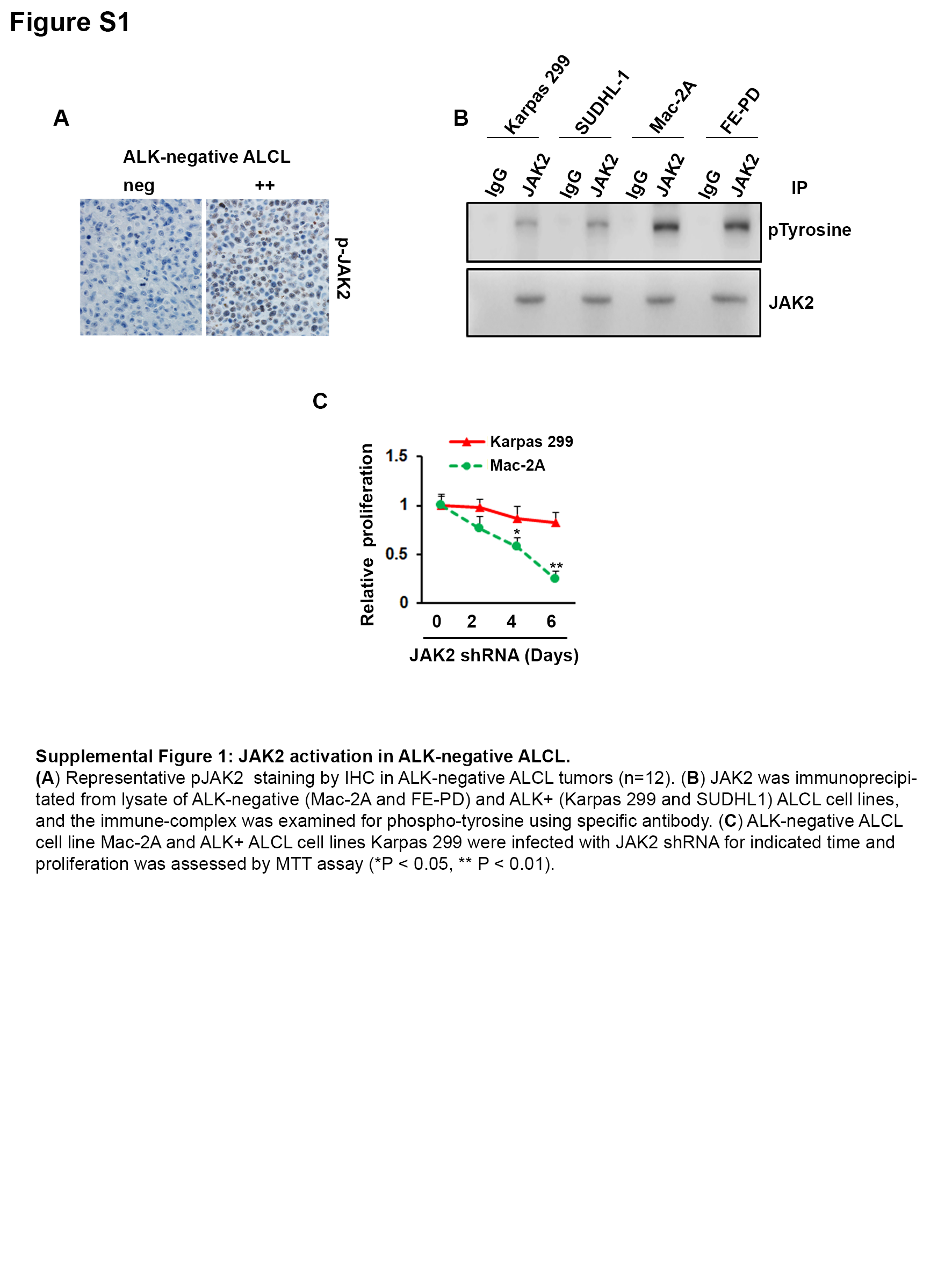

Supplement: Supplementary file 1 — Supplementary Figure 1 [file 41408_2021_447_MOESM1_ESM.tif]

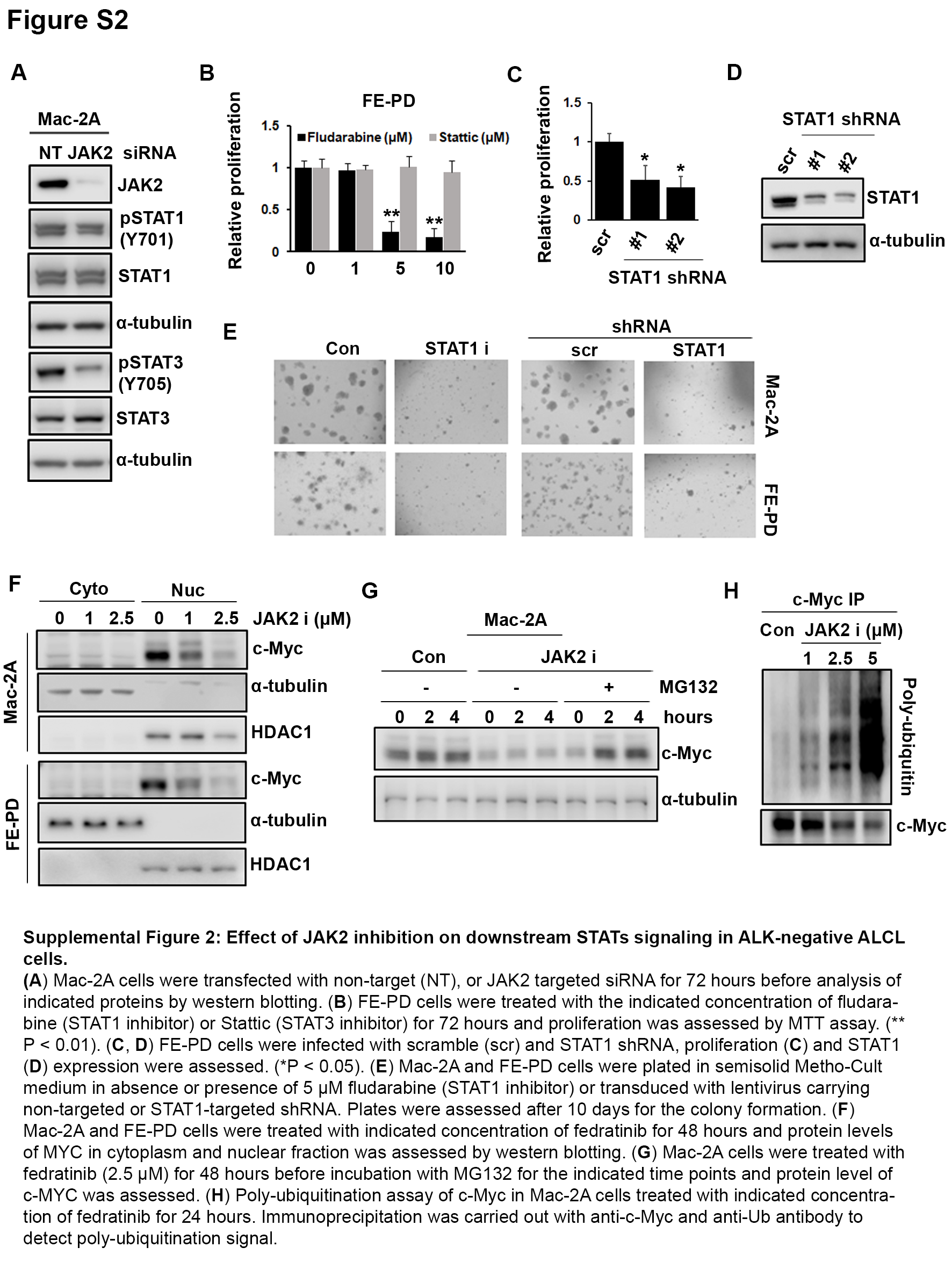

Supplement: Supplementary file 2 — Supplementary Figure 2 [file 41408_2021_447_MOESM2_ESM.tif]

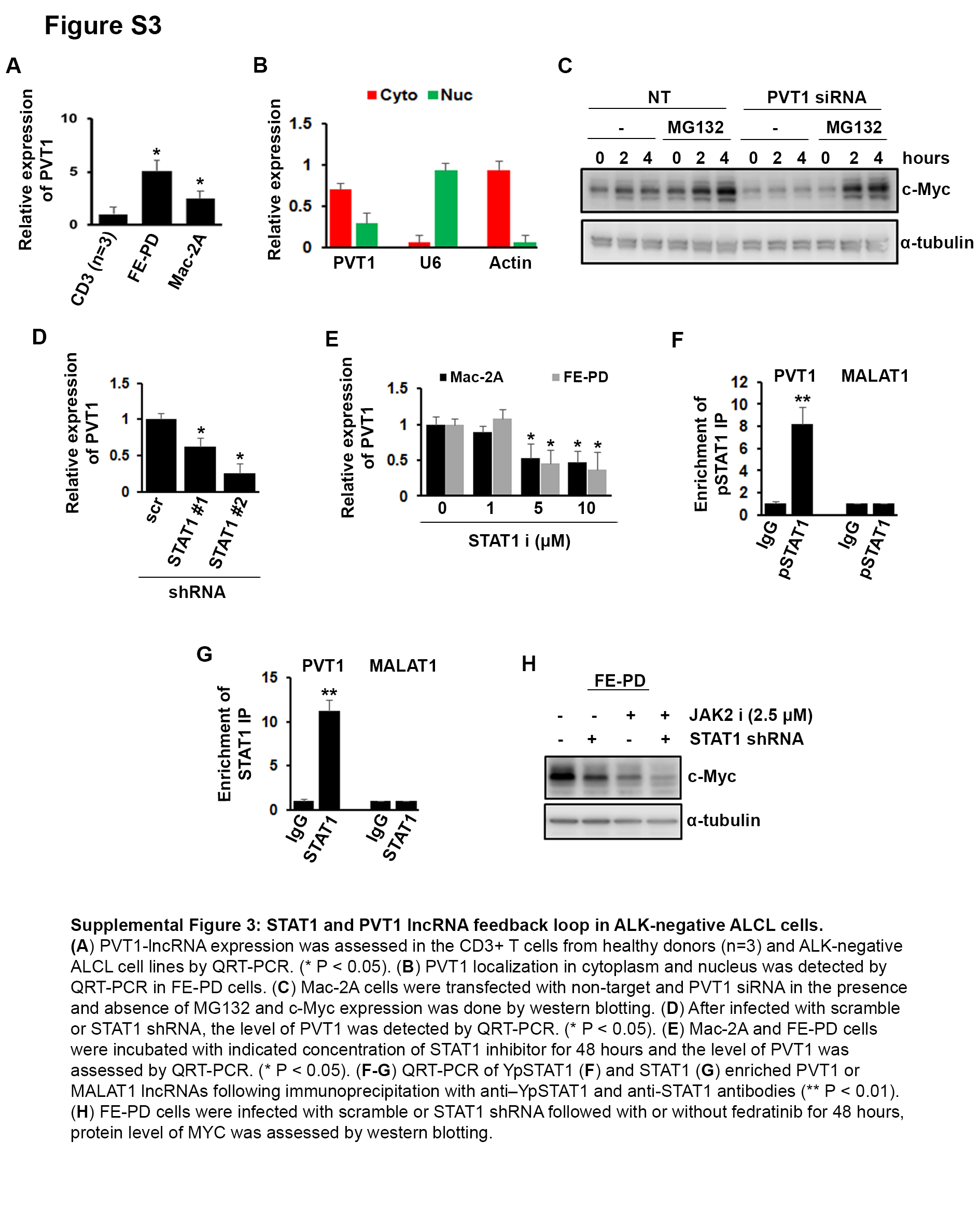

Supplement: Supplementary file 3 — Supplementary Figure 3 [file 41408_2021_447_MOESM3_ESM.tif]

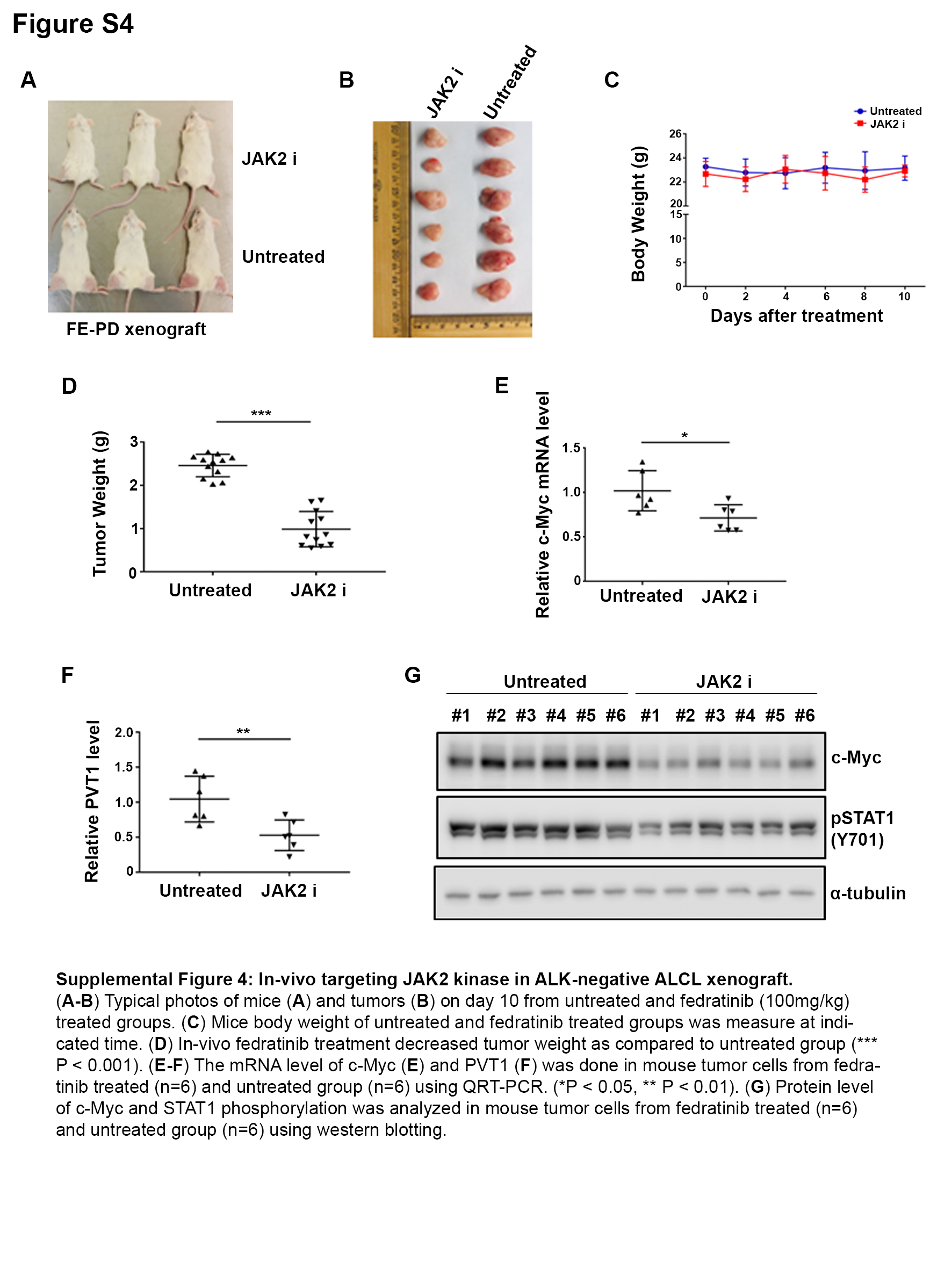

Supplement: Supplementary file 4 — Supplementary Figure 4 [file 41408_2021_447_MOESM4_ESM.tif]
